# Meeting Abstracts from the British Society of Breast Radiology Annual Scientific Meeting 2022

**DOI:** 10.1186/s13058-023-01659-8

**Published:** 2023-06-21

## Proffered Papers

### O1 The Epping Paperless Breast Screening Pathway—Designing, Testing and Implementing a Fully Paperless Screening Process

#### **Dr Anthony Aylwin**^**1**^, Helen Newman^1^

##### ^1^The Epping Breast Screening Unit, Princess Alexandra NHS Hospital Trust, Harlow, UK

*Breast Cancer Research* 25(S1): O1

As a unit, we implemented wireless image transfer from our Screening Mobile van in 2016, which resulted in some reduction in paper usage (only printing Client forms for those attending). However, the suspension of Screening at the start of the pandemic enabled us to undertake a multidisciplinary (radiological, radiographic and especially administrative) in-depth assessment of the existing pathway and design an equivalent electronic pathway, entirely without paper from screen to result. Redundant/duplicated paper-based processes (generally historical) were identified and expunged.

The key has been the utilisation of the NBSS whiteboard (SAWB) as a ‘nerve centre’ to allow tracking of clinics waiting to be closed, read, and reconciled, as well as those with outstanding results.

Processes were designed to allow.

- communication of clinical findings between mammographer and reader (using ‘Clinical Alert’ system on NBSS),
- verification of image checking on PACS (via a standalone database),
- recording of abnormalities on PACS images
- communication of assessment imaging requirements to the admin team.

Utilising a ‘dummy’ NBSS, the process was road-tested from invite to result, identifying and resolving all appreciable potential pitfalls, confirming the proof of concept.

It had a ‘hard go-live’ in November 2020 (due to losing the entire admin team to isolation), with the administration performed remotely, with complete success.

We have continued to date, with some modifications, and no major issues. However, further improvements would require changes to the NBSS platform (to which end we are currently working with Hitachi).

### O2 To investigate whether breast density is a predictor for subsequent events in patients with screen-detected DCIS, taking other factors into account

#### **Dr Nisha Sharma**^**1**^, Dr Mathew Wallis^2^, Bridget Hilton^3^, Karen Clements^3^, Prof Abeer Shaaban^4^, Prof Sarah Pinder^5^, Prof David Dodwell^6^, Prof Cliona Kirwan^7^, Dr Simon Lowes^8^, Dr Senthurun Mylvaganum^9^, Dr Janet Litherland^10^, Dr Elinor Sawyer^5^, Mrs Hilary Stobart^11^, Olive Kearins^3^, Dr Elena Provenzano^2^, Joanne Dulson-Cox^3^, Samantha Brace-McDonnell^11^, Prof Alastair M Thompson^12^

##### ^1^Leeds Teaching Hospital NHS Trust, Leeds, UK, ^2^Addenbrookes Hospital, Cambridge and Cambridge Breast Unit, and NIHR Cambridge Biomedical Research Centre, Cambridge University Hospitals NHS Trust, Cambridge, UK, ^3^NHS England and NHS Improvement, Birmingham, UK (pre-Oct 2021 Public Health England, UK),^4^Queen Elizabeth Hospital Birmingham and University of Birmingham, Birmingham, UK, ^5^ School of Cancer and Pharmaceutical Sciences, King's College London, Guy’s Comprehensive Cancer Centre at Guy’s and St Thomas’ Hospitals NHS Foundation Trust, London, ^6^Nuffield Department of Population Health, University of Oxford, Oxford, UK, ^7^Division of Informatics, Imaging & Data Sciences. School of Health Sciences, Faculty of Biology, Medicine and Health, University of Manchester, Manchester, UK, ^8^Gateshead Health NHS Foundation Trust, ^9^Royal Wolverhampton Hospital NHS Trust, Wolverhampton, UK, ^10^NHS Greater Glasgow and Clyde, Glasgow, UK, ^11^Independent Cancer Patients' Voice, London, UK, ^12^Baylor College of Medicine, Houston, Texas, United States

*Breast Cancer Research* 25(S1): O2

Aim: to investigate whether breast density is a predictor for subsequent events in patients with screen-detected DCIS, taking other factors into account.

Cohort and Exclusions: Patients entered into the Sloane trial with screen-detected DCIS (between 1 April 2003 and 31 March 2012), treated with breast-conserving surgery. Mastectomy patients and patients with LCIS were excluded. Follow-up period was just under 16 years.

Method: Multivariate Cox regression on breast density split into low (fatty and scattered fibroglandular) and high (heterogeneously and extremely dense) combined with other possible predictors of recurrence was performed. Analysis was carried out in R Studio (R version 4.1.2) for 4 separate models for subsequent events:

- Ipsilateral invasive
- Contralateral invasive
- Ipsilateral DCIS
- Contralateral DCIS

Results: The SLOANE database contains 11,644 cases of DCIS. 1655 developed subsequent events of which 1619 were malignant—1180 invasive and 439 non-invasive.

The subsequent event rate was 28% higher for high-density breasts than for low-density breasts when looking at ipsilateral invasive events. Density did not impact on subsequent contralateral invasive events or either type of DCIS events.

The model shows that after adjusting for other factors, having high density breasts increases the rate of ipsilateral invasive recurrence by 39% and receiving endocrine therapy reduces the rate by 37%.

Summary

Women with dense breast might preferentially benefit from anti-oestrogen therapy.

### O3 The impact of preoperative axillary assessment on surgical management of the axilla in screen-detected breast cancers

#### Dr Nisha Sharma^1^, **Dr Eleanor Cornford**^2^, Jackie Walton^3^, Olive Kearins^3^

##### ^1^Leeds Teaching Hospital NHS Trust, Leeds, United Kingdom, ^2^University Hospitals Plymouth NHS Trust, Retired, ^3^NHS England and NHS Improvement, Birmingham, UK (pre-Oct 2021 Public Health England, UK)

*Breast Cancer Research* 25(S1): O3

Objectives: To evaluate preoperative axillary assessment of patients treated with screen detected breast cancer in the NHS Breast Screening Programme.

Methods: In the 3-year period from April 2016 to March 2019, 6787/37349 (18%) patients with surgically treated invasive screen detected breast cancer in England had positive (malignant) nodes at surgery. Data were extracted from the National Breast Screening System (NBSS) to assess the accuracy of the assessment process including a comparison of fine-needle aspiration with core biopsy. Tumour characteristics affecting a positive preoperative biopsy were investigated along with operative management of patients with a positive preoperative nodal biopsy.

Results: 85.9% (31,438/3658) had an accurate preoperative diagnosis of nodal status at assessment. That is, the nodal status at assessment for 1493 node positive and 29,945 node negative at assessment was the same as the nodal status at surgery. (Sensitivity = 22.6%, Specificity = 99.9%).

For those patients subjected to biopsy, core had significantly higher sensitivity than FNA (81.2 vs 61.7%, *p* value < 0.00001).

Positive preoperative nodal biopsy was more likely in patients with higher nodal burden (*p* value < 0.00001).

88.9%of patients with a positive preoperative nodal biopsy had an axillary clearance as the first axillary procedure. 42% of this group had 2 or less positive nodes which may indicate overtreatment of the axilla.

Conclusion: Core biopsy has higher sensitivity and equivalent specificity for axillary biopsy. Imaging information from the axilla needs to be tailored to minimise overtreatment of the axilla in women with low axillary burden on imaging.

### O4 Non-Invasive Assessment of Breast Adipose Tissue with Quantitative MRI May Enable Detection of Cancer and Contribute to the Assessment of Cancer Risk

#### **Isobel Gordon**^**1,2**^, Dr George Ralli^2^, Dr Carolina Fernandes^2^, Dr Amy Herlihy^2^, Gemma Greenall^2^, Professor Sally Collins^1,2^, Professor Sir Michael Brady^1,2^

##### ^1^University of Oxford, Oxford, United Kingdom, ^2^Perspectum Ltd., Oxford, United Kingdom

*Breast Cancer Research* 25(S1): O4

Background: The composition of breast adipose tissue varies with disease state and is associated with cancer risk. A measure of breast fat composition is not currently provided in clinical practice; it is instead estimated indirectly through BMI.

Proton density fat fraction (PDFF) maps generated with non-contrast MRI have potential to directly characterise breast fat and can also enable calculation of breast density from a single non-invasive acquisition. Together, these two quantitative measurements could enable personalised risk assessment. Furthermore, identification of localised changes to breast fat composition could aid the detection of cancer.

**Methods:** Healthy women, women with breast cancer, and women with benign breast disease who gave informed consent underwent a two-minute MRI scan. Three-dimensional breast PDFF maps were generated using a breast-specific post-processing method (Gordon et al. 2022)^1^.

Results: In *N* = 35 healthy participants, the PDFF of breast adipose tissue correlated with BMI (*R* = 0.66, *p* < 0.0001). In two participants with histologically confirmed breast cancer, localised regions of lowered PDFF were found in proximity to malignant lesions. Such regions were not identified around benign lesions.^2^

Discussion: The association of breast adipose tissue PDFF with BMI could be reflective of adipocyte hypertrophy, which is associated with weight gain and thought to promote tumourigenesis. The reduction of adipose tissue PDFF around cancer could reflect the delipidation and browning of adipose tissue, or oedema.

Preliminary results show that breast adipose tissue PDFF corresponds to a known risk factor for breast cancer and may assist with cancer detection through characterisation of the adipose tumour microenvironment.

Acknowledgements: We gratefully acknowledge the support provided by the Royal Commission for the Exhibition of 1851 and Perspectum Ltd.

### O5 Long term breast cancer risk of B3 lesions managed with vacuum assisted excision—a ten year prospective review

#### **Dr Nerys Forester**^**1**^, Dr Hina Faisal^1^

##### ^1^Newcastle Teaching Hospitals NHS Trust, Newcastle, UK

*Breast Cancer Research* 25(S1): O5

Introduction: B3 breast lesions have a reported increased malignancy risk. Current management comprises vacuum-assisted excision (VAE) and 5-year mammography follow-up (atypical lesions). Limited published data for lesions managed this way. This prospective study addresses incidence, nature, timing of malignancy after B3 diagnosis.

Methods: Prospective review B3 lesions (no previous/concurrent malignancy) identified 01/12–12/16 for subsequent breast cancer, compared with group benign screen-detected lesions from 2012.

Results: 514 B3 lesions (RS/CSL (22.2%), AIDEP (21.4%), papilloma (21.8%), LN (18.3%), FEA (8.4%), misc (5.3%)). 391 underwent VAE; 36 upgraded to B5, 18 upgraded following diagnostic excision (upgrade 10.5%). Remaining 460 patients had follow-up; mean of 3.4 mammograms (range 0–8), 44 no further mammograms (age/personal choice), 14 deaths (1 breast cancer).

Thirty-three women (7.2%) developed breast cancer; 23 identified by mammography. One lymphoma detected by mammography. Median time-to-diagnosis was 4 years (range 1–11 years). Ten cancers at the site of the initial B3 lesion. No significant difference between cancer development in B3 subgroups observed.

8/147 (5.5%) women with benign lesions (B2/C2 pathology) developed cancer. No significant difference between B3 lesions or controls at 5 years identified.

Discussion: 7.2% of women with B3 lesions subsequently develop breast cancer. Cancer occurred at original B3 site in one-third and was screen-detected in approximately two-thirds of cases. Rate of cancer development was not significantly higher than that in screened controls. Safe follow-up strategy of B3 lesions could comprise mammographic review at one year followed by return to routine breast screening.

## Posters—Alphabetical by presenting author

### P1 Evaluation of impact of addition of Digital Breast Tomosynthesis (DBT) in diagnostic workup of breast lesions of uncertain malignant potential (R-3)

#### **Dr Nadia Akhtar**^**1**^

##### ^1^Northampton General Hospital NHS Trust, Northampton, England

*Breast Cancer Research* 25(S1): P1

Materials and Methods: This single-centre, retrospective and quantitative analysis enrolled 385 women recalled from NHSBSP and symptomatic breast clinics between July 2020 and December 2021. They had DBT performed. BIRADS grading and lesion size on FFDM and DBT were compared using a paired T-test. The relationship between breast density and change of BIRADS scoring along with change in lesion characterisation and mean absolute size deviation for FFDM and DBT from histopathological size was shown using Pearson’s correlation test. Receiver operating characteristic (ROC) curves and area under the curve (AUC) were calculated for FFDM + DBT and FFDM alone.

Results: 309/355(87%) of BIRADS-3 lesions on FFDM changed their BIRADS classification after the addition of DBT. One hundred and fifty-one (42.5%) cases were confirmed normal and 78(20.5%) cases were confirmed benign after DBT. All cases graded as BIRADS-5 on DBT and 39/41 cases graded as BIRADS-4 on DBT were confirmed cancers on biopsy. Change in lesion BIRADS grading after DBT was seen across all the breast densities. Change on BIRADS grading and lesion characterisation after DBT was more pronounced in dense breasts. Pearson’s correlation coefficient showed mean absolute size deviation from histopathological size was larger for FFDM (5.34 Mm) (*p* < 0.001) as compared to DBT (2.49 mm) (*p* = 0.019). Sensitivity, PPV, NPV and diagnostic accuracy of DBT are better than FFDM. Comparison of AUC of two modalities shows that DBT has significantly greater AUC than FFDM (*p* < 0.001).

Conclusion: detection of true-positive cases increases with FFDM + DBT than FFDM alone. There is better concordance with BIRADS grading and lesion characterisation by DBT and results of histology. The size measurements are more accurate with DBT than with FFDM.

### P2 Can the Enhancement Gradient of a Lesion Between Early and Delayed Phase Contrast Enhanced Spectral Mammography Imaging Predict Histology?

#### **Dr Anam Ali**^**1**^, Dr Linda Metaxa^1^, Dr Tamara Suaris^1^

##### ^1^St Bartholomews Hospital, London, United Kingdom

*Breast Cancer Research* 25(S1): P2

Purpose: the aim was to evaluate the enhancement gradient of histologically different breast lesions between early- and delayed-phase CESM imaging.

Methods: A retrospective analysis of 52 patients undergoing CESM at St Bartholomew’s Hospital (November 2020–2021) was performed. Lesions with histologically proven diagnoses were included. Degree of enhancement on CESM was quantitatively assessed by measuring the region of interest (ROI) signal difference between the lesion and background parenchyma, to obtain a percentage ROI signal difference (%RS), on both CC and MLO views. This was done for all lesions on both early- and delayed-phase images. The difference in %RS between the delayed- and early-phase CESM imaging was used to evaluate an enhancement gradient. Statistical analysis was carried out to determine whether this gradient correlated to histology.

Results: 95 lesions analysed; 43 (45.2%) invasive cancers, 26 (27.4%) non-invasive cancers, 26 (27.4%) benign lesions. Lesions showed different enhancement gradient patterns (*p* < 0.01) between early- and delayed-phase CESM imaging. Invasive cancers showed a decreasing enhancement gradient (Mean %RS − 0.16%); however, the reduction in enhancement was not statistically significant either in MLO (*p* = 0.4, 95% CI +/− 0.4) or in CC views (*p* = 0.2, 95% CI +/− 0.36). Non-invasive cancers (Mean %RS + 0.19%) and benign lesions (Mean %RS + 0.21%), demonstrated an increasing enhancement gradient, which was statistically significant for the non-invasive cancers (*p* = 0.03, 95% CI +/− 0.3) and showed a distinct trend towards significance in benign lesions (*p* = 0.07, 95% CI +/− 0.2).

Conclusions: Histologically different breast lesions have different enhancement gradient patterns between early- and delayed-phase CESM imaging.

### P3 Accuracy of breast MRI in detecting presence of residual disease after neoadjuvant chemotherapy

#### Dr Lyndsey Brierley^1^, Dr Naveed Altaf^1^, Dr Aninda Saha^1^, **Dr Anuradha Anand**^**1**^

##### ^1^University Hospital of North Tees and Hartlepool, Stockton-on-Tees, United Kingdom

*Breast Cancer Research* 25(S1): P3

Background: Breast MRI performed before and after neoadjuvant chemotherapy (NACT) aims to identify and quantify residual disease and thereby aid surgical planning.

Method: Pre- and post-NACT MRI reports were compared with final surgical histology to assess the accuracy of MRI in predicting the presence or absence of residual breast and axillary disease.

Results: (1) Out of 60 patients, 19 achieved complete pathological response (PCR) with no residual invasive disease or DCIS. MRI correctly predicted 16 /19.

5/60 had PCR with residual DCIS, which in our unit is considered to be PCR. Of these 5, MRI predicted complete response in 2 and identified residual disease in 3.

36/60 patients had residual disease. This was correctly identified by MRI in 27 cases. Of the remaining 9 cases, 7 demonstrated less than 10% residual disease in the tumour bed.

If DCIS is considered residual disease, sensitivity of MRI in detecting residual invasive disease or DCIS = 73%, Specificity = 84%, Accuracy = 77%, PPV = 91% and NPV = 59%.

If only invasive cancer is regarded as residual disease and the presence of DCIS is classified as PCR (as in most units across the UK), Sensitivity = 75%, Specificity = 75%, Accuracy = 75%, PPV = 82% and NPV = 67%.

(2) Out of 24 patients with biopsy-proven nodal metastases, 14 had no residual cancer at axillary surgery. MRI predicted 9/14. Ten patients had residual axillary disease, which was correctly predicted by MRI in 5/10 cases. Sensitivity of MRI in predicting residual axillary disease = 50%, Specificity = 64%, PPV = 50% and NPV = 64%.

Conclusion: These results are in line with published literature.

### P4 Audit of accuracy of iodine seeds in the pre operative localization of breast cancers

#### Dr Sarrah Eltahir^1^, Dr Tiffany Lau^1^, Dr Naveed Altaf^1^, Dr Aninda Saha^1^, Matei Dordea^1^, **Dr Anuradha Anand**^**1**^

##### ^1^University Hospital of North Tees and Hartlepool, Stockton-on-Tees, United Kingdom

*Breast Cancer Research* 25(S1): P4

Background: Our Trust has been using Radioactive iodine seeds for the preoperative localisation of non-palpable breast cancers since 2018. After achieving excellent results in localising ultrasound visible mass lesions (6% re-excision rate using seeds versus 13% using guidewires) and following our ARSAC licence extension to allow stereotactic guided iodine seeds, we extended the use of iodine seeds to all preoperative localisations, i.e. stereotactic localisation of calcification and B3 lesions. This audit was performed to assess the accuracy of iodine seeds in preoperative localisation of all lesions requiring wide local excision.

Method: This audit reviewed all iodine seed insertions in our trust between 01.12.21 and 20.10.21. We reviewed the final surgical pathology results to ascertain completeness of excision and need for re-excision.

Results: Of a total of 206 cases, 157 lesions were completely excised and 49 had incomplete excision.

Re-excision was required in 27 cases (13%).

All the 27 cases requiring re-excision contained DCIS. Thirteen demonstrated non-calcified DCIS, 6 demonstrated calcified DCIS on pathology but no corresponding calcification on preoperative imaging, 8 demonstrated calcified DCIS on both imaging and pathology.

Conclusion: Our overall re-excision rate has improved from 17% prior to the introduction of Iodine seeds to 13% after the introduction of seeds, despite the initial learning curve for Radiology and Surgery.

All cases requiring re-excision contained DCIS, and 70% of these had no visible calcification on mammography, indicating that radiologically invisible disease is a crucial factor in underestimating disease extent.

### P5 A pictorial review of retroareolar cancers and cancer mimics

#### **Dr Maria Asad**^**1**^, Dr Farah Walajahi^2^, Dr Trupti Kulkarni^2^

##### ^1^Wythenshawe Hospital, Manchester University NHS Foundation Trust, Manchester, United Kingdom, ^2^Nightingale Centre, Wythenshawe Hospital, Manchester University NHS Foundation Trust, Manchester, United Kingdom

*Breast Cancer Research* 25(S1): P5

Background: Retroareolar cancers account for 8% of breast cancers. Radiologically, assessment of this anatomical region is challenging. Imaging findings may be subtle and careful evaluation with a high index of suspicion is needed. Imaging is also essential for guiding biopsy for tissue diagnosis.

Summary: The retroareolar region is the area within two cm from nipple–areola complex. Despite its superficial location, ultrasound assessment can be difficult due to artefacts such as acoustic shadowing, which can mimic or mask a mass.

Mammograms used for screening asymptomatic women may show very subtle changes such as minimal change in shape or orientation of the nipple and indentation of the areola. Sensitivity of mammography in the retroareolar region is further reduced in the presence of dense tissue in this location which may mask a small mass.

Learning Points: We aim to highlight the pitfalls in assessing this challenging but important area through a series of cases demonstrating findings of retroareolar cancers and cancer mimics.

### P6 Wireless localisation at assessment—Pilot study in a large screening centre

#### **Dr Gauri Babu**^**1**^, Janet Clarke^1^

##### ^1^South East Scotland Breast Screening, Edinburgh, UK

*Breast Cancer Research* 25(S1): P6

Aim: We performed a pilot study with wireless localisations using Saviscout reflector at our screening centre. This was done to establish the benefit of localisations at assessments to improve patient pathway. We are a large screening centre with several regional hospitals; however, surgery was performed for the screening population at a single symptomatic unit. Trial to improve patient pathway and allow repatriation to the regional centres for surgery by placement of localisers at point of assessment.

Methodology: 25 reflectors were placed as part of this pilot. The patients were identified on day of assessment. Unifocal, non-palpable cancers or separate cancers in BIRADS 1 density were deemed suitable. A patient leaflet was designed, and written consent obtained. The suitability was established as U5 on ultrasound and scout placed after biopsy instead of a marker. We confirmed deployment with a detector probe and check mammogram. An online patient satisfaction questionnaire was distributed.

Results: Of the 25 cases, tumour sizes ranged from 7 to 23 mm with maximum depth from skin up to 16 mm. The re-excisions rates were minimal in this group.

Conclusions: The availability of this technique significantly improved our patient pathway from screening assessment to surgery. We are in the process of repatriating the surgery to regional units, thereby improving the accessibility for patients to be treated in their regional hospitals and reduce the need for travel during the pandemic. The patient experience recorded showed satisfying results too alongside significant cost savings for the trust.

### P7 Retrospective study on Savi–Scout Localisation—A DGH Experience

#### **Dr Soumya Baxi**^**1**^, Dr Ben Ward^2,3^, Dr Apurva Sinha^1,2^

##### ^1^University of Edinburgh, Edinburgh, Scotland, ^2^Borders General Hospital, Melrose, Scotland, ^3^NHS Lothian Screening Service, Edinburgh, Scotland

*Breast Cancer Research* 25(S1): P7

Background: Wire-guided localisation (WGL) has been the preferred method of localisation for non-palpable breast lesions. Limitations with WGL have led to the development of various other methods of localisation techniques. We adopted the Savi-seed as the preferred method of localisation in our DGH setting with the aim of improving the patient journey and saving both radiological and surgical time.

Materials and methods: Retrospective study of patients undergoing Savi-seed deployment over the period of 5 months for non-palpable breast cancer.

Results: Of the 23 patients undergoing therapeutic excision, all presented at the symptomatic clinic. Savi-seed was either deployed at the time of the initial clinic appointment (n-4), or electively after discussion at the MDT (n-19). All patients either had either excellent or satisfactory deployment post-insertion with no immediate post-procedural complication or failure.

Discussion: Our early experience in a DGH confirms the safety and effectiveness of this procedure in improving the patient journey. After an initial trial, we now routinely deploy the Savi-seed in all impalpable unifocal cancer (R5/U5 on conventional imaging) during the patient’s first symptomatic clinic attendance.

With the ability to deploy the reflector there have been fewer delays on the day of surgery, flexibility in planning surgical lists and minimal discomfort associated with deployment. It appears that there is an overall cost benefit with the procedure by saving both radiological and surgical time and resources. We are planning to study the cost-effectiveness of the procedure (compared to WGL), which would further re-enforce the overall effectiveness of this procedure.

### P8 An audit on MRI-guided breast biopsies in mammographic and ultrasound-occult breast lesions

#### **Dr Basrull Bhaludin**^**1**^, Dr Dione Lother^1^, Dr Kate Downey^1^, Dr Tanja Gagliardi^1^

##### ^1^The Royal Marsden NHS Foundation Trust, London, United Kingdom

*Breast Cancer Research* 25(S1): P8

Aim: The aim of this analysis is to identify the incidence of cancers from a series of MRI-guided breast biopsies performed at the Royal Marsden. We also aim to correlate the MRI findings with histopathological outcomes of biopsies and compare our performance against the positive predictive malignancy rate of MRI-guided breast biopsy outlined by the ACR BI-RADS Atlas 2013. Due to the high cost and time required to do these procedures, assessing our performance will indicate whether this service is valuable to our institution and the referring centres within our network.

Methods: A retrospective analysis of MRI-guided biopsies performed over a 12-month period at the Royal Marsden was undertaken. Imaging and pathology records were obtained using EPR and PACS.

Results: There were a total of 66 patients who underwent MRI-guided biopsies between April 2021 and April 2022, with 69 lesions sampled in total.

Out of these, there were 14 invasive cancers (B5b), 14 in situ cancers (B5a) and 1 highly suspicious pathology (B4). Eight were B3 lesions and 32 were B1/B2 results.

Discussion: We identified a 41% incidence of breast cancers within our retrospective analysis, which is in line with the 20–50% benchmark positive predictive malignancy rate of MRI-guided breast biopsy outlined by the ACR BI-RADS Atlas 2013. The results indicate that this service is valuable to our institution and external referrers in our network.

### P9 Pre-operative assessment of the axilla in breast cancer: changing the threshold

#### **Dr Vishal Chohan**^**1**^, Sophie James^1^, Michael Rees^1^, Dr Karene Lim^1^

##### ^1^Aneurin Bevan University Health Board, Newport, United Kingdom

*Breast Cancer Research* 25(S1): P9

Objectives: Ultrasound of lymph node size at the axilla is an important investigation in the assessment of peri-operative breast cancer. At present, there are no definite guidelines to determine the threshold to biopsy a node based on its cortical thickness. A change in measurement from 2.5 mm to 3 mm was recently introduced at our centre. Retrospective analysis was used to determine the validity of this implementation.

Method: Patient demographics, preoperative axillary ultrasound findings and post-operative histology results were obtained for patients who underwent an axillary node clearance between 10/2018 and 09/2021.

Results: The data of 98 patients were analysed [median age = 62 years; 96 (98%) female]; median lymph node cortical thickness was 4.2 mm (1.25–50 mm). The sensitivity for preoperative detection of lymph node metastases with axillary ultrasound was 93% (*n* = 91) using the existing cortical thickness threshold of 2.5 mm. With a higher cortical thickness threshold of 3 mm, the estimated sensitivity for preoperative detection of lymph node metastases was 84% (*n* = 82, *y*2 = 0.23, *p* = *n*). Axillary lymph node metastases were found in 18 patients (13%) where the lymph node cortical thickness was < 2.5 mm. In patients who were heavily node positive (> 4 nodes/N2 disease, *n* = 50), the sensitivity of the technique improved to 94% (*n* = 47) independent on whether the cortical thickness biopsy threshold was 2.5 or 3 mm.

Conclusion: A small yet non-significant effect to detect pathological nodes was appreciated by increasing the threshold from 2.5 to 3 mm. No difference was observed in patients with heavy nodal disease. Prospective data will be used to substantiate these findings.

### P10 5 years of experience in Pregnancy Associated Breast Cancer (PABC)—A pictorial review of radiological features

#### Dr Austin Donnelly^1^, Dr Elaine Davis^1^

##### ^1^Belfast Health and Social Care Trust, Belfast, N Ireland

*Breast Cancer Research* 25(S1): P10

Learning objectives

1. Highlight that although uncommon, pregnancy-associated breast cancer (PABC) is the second most common malignancy detected during pregnancy.
2. Illustrate the important imaging features in PABC.
3. Highlight that often, these cancers are mammographically occult due to physiological increase in breast density during pregnancy.
4. Stress the need for a high index of suspicion and the important role that other imaging modalities play in detection of PABC

Background: Pregnancy-associated breast cancer (PABC) is defined as any breast carcinoma diagnosed during pregnancy or during the first postpartum year. Estimates of frequency range from 1 in 1500—1 in 10,000 pregnancies. PABC is associated with poorer outcomes than non-PABC with a 5-year and 10-year survival of 52.1% and 43.9%, respectively (as compared to 80% and 68.6% 5 and 10-year survival rates in matched, non-PABC cases).

Imaging findings: We present a series of 11 cases of PABC from our institution that demonstrate the diagnostic imaging features on ultrasound (US), mammography and magnetic resonance imaging (MRI).

Specifically, we highlight the wide range of radiological appearances in PABC and illustrate that many cases are mammographically occult.

We demonstrate the use of contrast-enhanced mammography as an adjunct to standard mammography.

Conclusion: Although rare, PABC is the second most common malignancy detected during pregnancy. A high index of suspicion and often multimodality investigation is required to ensure timely diagnosis leading to better patient outcomes.

### P11 Accuracy of Contrast Enhanced Spectral Mammography vs Breast MRI in assessing invasive lobular breast cancer

#### **Dr Elliot Elwood**^**1**^, Dr Ambika Kapoor^1^, Dr Priyanka Belaguthi^1^, Dr Tharsi Sarvananthan^1^, Dr Philippa Skippage^1^, Dr Kirsten Stafford^1^, Dr Emily Daulton^1^

##### ^1^Frimley Health NHS Foundation Trust, Farnborough, United Kingdom

*Breast Cancer Research* 25(S1): P11

Background: Invasive lobular breast cancer's (ILC) elusive nature on standard breast imaging poses a diagnostic challenge. Currently, breast MRI is the gold standard imaging tool for assessing ILC. Emerging tools such as contrast-enhanced spectral mammography (CESM) utilise similar neovascularity principles of tumour enhancement to those of MRI. We aim to evaluate the clinical utility of CESM vs. Breast MRI for assessing ILC.

Method: Retrospective single-centre analysis of all biopsy-proven ILC who underwent MRI and CESM between April 2018 and December 2021. The diagnostic performance of CESM and MRI was evaluated using final surgical histopathology. Sensitivity and positive predictive values were calculated. Size concordance was analysed utilising paired t-tests.

Results: A total of 96 lesions were identified of which 69 lesions represented primary index tumour with additional 27 multifocal areas. Of the primary index lesions, 13% were not detected on CESM (9/69), whereas all were seen on MRI. Of the additional multifocal enhancing areas on MRI, 33% (9/27) were negative on CESM. Thus, sensitivity for detecting multifocality was enhanced with MRI than CESM (100% vs. 82%). PPV was comparable for both modalities (90% for CESM and 89% for MRI). Size concordance analysis demonstrated CESM underestimates size compared to MRI with a mean difference of − 4.6 mm and − 3.3 mm, respectively.

Conclusion: Our results demonstrate better sensitivity of MRI compared to CESM in detection of multifocal disease and accuracy of size evaluation in ILC. CESM, however, provides a useful adjunct where there is diagnostic uncertainty and suitable alternative where MRI is contraindicated.

### P12 Preventing unnecessary referrals for incidental breast lesions detected on cross-sectional imaging; a review and audit of a local referral pathway

#### **Dr Rida Fatima**^1^, Dr Lara Jehanli^1^, Dr Sheetal Sharma^1^

##### ^1^Royal Liverpool and Broadgreen University Hospitals NHS Trust

*Breast Cancer Research* 25(S1): P12

Background: Increased use of cross-sectional imaging has resulted in greater detection of incidental breast lesions. There are no guidelines for how these findings should be followed up.

Prior to the implementation of our pathway, local practice was for general radiologists to refer incidental breast findings either to symptomatic breast clinic or to breast MDT.

This referral pathway allows general radiologists to refer patients with incidental breast findings to a breast radiologist (by adding a JBREAS code in the report) who can then review the imaging. The breast radiologist can then decide whether symptomatic clinic referral is appropriate and communicate this via an addendum to the original report.

Aim: Review all scans with JBREAS code since 2015, to assess whether the system is effective in avoiding unnecessary referrals.

Methods: Retrospective review of scans with JBREAS code between 27/11/2015 and 07/12/2021. Analysis of patient data using CRIS and ICE for patient and scan details and histology results.

Results: Out of a total of 391 scans, 194 patients were recommended for breast clinic referral. Forty-seven of those referred to clinic had malignant histology.

Discussion: The data show that our pathway reduced referrals by 50%. This significantly reduced the burden on oversubscribed breast clinics.

Advantages to patients include saving unnecessary anxiety and trips to the hospital, thereby reducing travel costs and environmental impact.

Furthermore, the pathway enabled rapid assessment of incidental breast lesions that were subsequently found to be malignant in nature (24% of total JBREAS referrals).

### P13 Arbitration/consensus meeting, is it worth it?—A symptomatic breast unit experience

#### **Dr Rosanna Frost**^**1**^, Dr Diana Dalgleish^1^, Dr Helen Burt^1^, Dr George Devenish^1^

##### ^1^Royal United Hospitals Bath, Bath, UK

*Breast Cancer Research* 25(S1): P13

Double reading screening mammograms are the current UK and European standard. This increases cancer detection rates whilst reducing recall rates, impacting on patient’s anxiety and have significant cost implications for time and resources. This practice, however, inevitably results in discordant results amongst readers, requiring resolution. To minimise our recall rates at our symptomatic centre, we discuss concordant and discordant recalls at a weekly group arbitration/consensus meeting. We audited these cases over an 18-month period.

Method: Between October 2020 and April 2022, patients discussed at the arbitration were audited. Data collected, included source of referral, mammographic reason for discussion, 1st and 2nd read M-classification, outcome of arbitration and outcome of assessment (benign, malignant, B3).

Results: 395 patients were discussed, out of 2673 mammograms performed (14%). Of the 395 patients discussed, 183 were either discharged or returned to routine recall. Our recall rate was 8% with 212 patients recalled for assessment. Of those, 51/212 (24%) patients had a malignancy detected. Out of 212, 151 (71%) had a benign or normal outcome from assessment. Ten (5%) patients returned a B3 result. Seven patients with a malignant outcome were recalled by a single reader (13%).

Conclusion: Our results show that our arbitration process results in significant reduction (46%) in the number of recalls. The practice of double reading whilst having significant impact on resources prevented the false negative reporting of 13% of the malignant outcomes. Further audit and research should be undertaken to establish national standards for double reading of mammograms undertaken in a symptomatic setting.

### P14 Prone and Supine Quantitative MRI Measurement of Breast Density: Preliminary Results from Ongoing IMOGEN Study

#### **Dr Amy Herlihy**^**1**^, Miss Grace Tiedeman^1^, Isobel Gordon^1,2^, Professor Sally Collins^1,2^, Professor Michael Brady^1,2^

##### ^1^Perspectum Ltd., Oxford, Oxfordshire, ^2^University of Oxford, Oxford, Oxfordshire

*Breast Cancer Research* 25(S1): P14

Introduction: Breast density has been identified as the single most important phenotypic risk factor for breast cancer in postmenopausal women and decreases mammography sensitivity where “density” is equated to radiological attenuation. Accurate quantification of dense breast tissue is important for risk stratification and patient management. Breast density is typically assessed qualitatively, resulting in high inter-/intra-observer variability. Quantitative MRI methods measuring breast density assign voxels as either fatty or fibrous tissue, though generally have low resolution. Measurement of breast density through MRI-PDFF maps^3^ can account for the mixed nature of some voxels. The woman is imaged prone, which many find uncomfortable.

We report a system to measure breast density on non-specialist MR systems, while the woman is supine.

Methods: Eighteen healthy participants were scanned prone and supine, generating 3D breast PDFF maps (Gordon et al.)^4^. Supine, the breasts were supported to avoid distortion by the RF coil. Breast density was calculated from the PDFF map. The agreement between the measures was assessed using Bland–Altman analysis and coefficient of variation (CoV).

Results: Prone and supine measures demonstrated: 95% limits of agreement − 6 to + 11%, with minimal bias of 3%; average coefficient of variation was low at 4.6%.

Conclusions: Breast density calculated supine has excellent agreement with prone, rendering the measurement more scalable to sites without specialised MRI coils. The protocol and breast support can be added straightforwardly to standard abdominal or thoracic scans, enabling breast density to be measured routinely as part of standardised acquisition protocols.

### P15 A review of the direct access to mammogram program introduced in Ireland for women over 35 years with breast pain

#### **Dr Michelle Horan**^**1**^, Dr. Sylvia O’Keeffe^1^

##### ^1^St James’ Hospital, Dublin 8, Ireland, Dublin 8, Ireland

*Breast Cancer Research* 25(S1): P15

Introduction: Breast pain, or mastalgia, is a frequent cause of referral to symptomatic breast clinics. It has a reported incidence of 52% but is infrequently associated with breast cancer (0–2.3%). In 2021, a direct-access program to mammography was introduced in Ireland for women over 35 years with mastalgia to avoid the need for clinical review.

Methods: A retrospective review of all women referred for direct access mammography with mastalgia during a six-month period from November 2021 to May 2022 was undertaken. Patient demographics, family history status, site of pain and details of the patients’ symptoms from the GP referral proforma were recorded. Mammography score, need for further imaging/biopsy and number diagnosed with a malignancy were determined.

Results: 372 patients were identified for inclusion (mean age 50.99, median 48, IQR 42–57). 6.9% (26) were recalled for further imaging with 4.3% (16) undergoing biopsy. Biopsy results: one B1, ten B2, one B3 and four B5 (1% of cohort). Of the four malignancies diagnosed, two were in the symptomatic breast with two in the contralateral breast. The women diagnosed ranged from 51 to 80 years old. Out of 372, 63 (16.9%) of the GP referrals did not document any pain.

Conclusions: The results of our review suggest a high cancer detection rate (CDR) of 10 per 1000 in women referred directly for mammography with mastalgia. This program was introduced during the COVID-19 pandemic when there was an almost complete cessation of breast screening, and therefore, CDR may have been falsely elevated by the absence of available screening mammography.

### P16 Predicting pathological axillary lymph node status on MRI breast following NACT for breast Cancer

#### Dr Amanjot Karuppiah^1^

##### ^1^Nottingham University Hospitals NHS Trust, Nottingham Breast Institute

*Breast Cancer Research* 25(S1): P16

This work was done as an audit project to aid service improvement.

Audit Standard: Baseline (Pre NACT) AUS Sensitivity 49–87% in literature—our unit High 70 s. Specificity 53–97%—our unit high 90’s.

Method: TOTAL included patients 44, all node positive at baseline, post-NACT, 2 MRI scans at least, underwent surgery-WLE/Mastectomy selected from oncology database 2020–2021. Final MRI and pathology reviewed to assess CRR in breast and axilla, CPR in Axilla.

Question to be answered-Can MRI breast be used to predict nodal status post-NACT in previously node positive patients?

Results: 14/ 44 cases showed complete radiological response both in breast and axilla on MRI 0.22 had partial radiological response in breast, 3 showed disease progression. Eighteen showed complete radiological response in breast on final MRI, 1 had MRI occult disease in the breast. Out of 18, 10 had complete Radiological response in the breast and complete pathological response in the breast.

SENSITIVITY of breast MRI (TP/TP + FN) 14/28 = 50%

SPECIFICITY of Breast MRI (TN/TN + FP)14/16 = 87.5%

Conclusion: MRI breast has comparable sensitivity to AUS 50%. This improves if there is CRR in both axilla and breast = approx. 71%. MRI breast has a comparable specificity to AUS 87.5%.

Action: Use MRI breast images to F/U axillary response to aid de-escalation of axilla in conjunction with clipping of nodes at initial biopsy.

Prospective reaudit in 1 year after implementation.

### P17 Breast screening MRI in women of high-risk: A pictorial review of assessment recalls in 2020 at St George’s Rose Centre

#### **Dr Sharon Koo**^**1**^, Dr Derin Khafaf^1^, Dr Sarah Mcwilliams^1^

##### ^1^St George’s University Hospital, London, United Kingdom

*Breast Cancer Research* 25(S1): P17

Breast MRI is the main imaging modality in Very High Risk Breast Screening due to its increased sensitivity in breast cancer detection compared to mammography alone and its ability to screen women under 40 without involving X-rays.

The limited specificity of breast MRI has a significant false-positive rate generating an increased recall rate, additional imaging, biopsies and patient anxiety. The balance between the recall rate and detection rate is challenging with the limited accessibility to MRI-guided biopsy and the limited number of clinicians interpreting breast MRI.

We analysed 24 patients recalled to assessment at St George’s from the Very High Risk Breast Screening Program in SW Thames in 2020 and present a pictorial review.

All patients were screened with annual MRI and/or mammography according to guidelines. The age range was 31–65 and included genetic deletions in BRCA1/2, PALB2, Cowden, Peutz Jeghers syndrome and previous mantle radiotherapy. Of the 24 recalls, 5 were cancers, 2 were clinical recalls, 10 yielded benign and 7 yielded normal findings.

The typical appearances of breast cancer in patients with genetic deletions, the use of early recall or MR biopsy and the complexity of reviewing screening modality at age 50 are illustrated. The challenges screening very-high-risk patients are discussed, including hormonal variations in background enhancement, and the difficulty maintaining round length due to pregnancy and breastfeeding.

In conclusion, Very High Risk Breast Screening is a challenging subgroup of women with up to a 40% lifetime incidence of breast cancer.

### P18 MRI Breast? Don’t forget the rest!

#### **Dr Karen Litton**^**1**^, Dr John Gifford^1^, Dr C Brolun-Napier^1^, Dr M Kimura^1^, Dr Nicholas Ridley^1^

##### ^1^Great Western Hospitals NHS Foundation Trust, Swindon, United Kingdom

*Breast Cancer Research* 25(S1): P18

MRI of the breast is increasingly used for the assessment and local staging of breast cancer as well for the screening of very-high-risk patients in the NHS breast screening programme.

A dynamic contrast-enhanced protocol is used as well as other sequences. These sequences assess the inherent features of the breast parenchyma and how gadolinium contrast affects this over time.

There is information outside of the breast available to us, and this should not be overlooked. Multiple sequences can give us clues to characterise unexpected findings.

We describe six review areas outside the breast where incidental finding should be sought.

1. Soft tissues, skin and muscle. Involvement by the primary tumour may influence surgical management. Skin thickening, oedema and intramuscular masses may represent advanced disease.
2. Axillae and other nodal stations. We describe less common sites of nodal disease, including internal mammary chain, inter- and sub-pectoral, epicardial and mediastinal nodes.
3. Bone marrow. The ribs, sternum and proximal humeri are often included and can be interrogated.
4. Great Vessels. Incidental thoracic aneurysms may require further screening or urgent referral.
5. Pericardium and Pleura. Abnormal fluid in these spaces may represent metastatic disease or other pathology.
6. Liver, Lungs and upper abdomen. Abnormalities can be found distantly. Some findings (i.e. liver cysts) can be called benign. Other findings may require further imaging.

By reviewing these areas, the radiologist may find additional unexpected pathology or important findings that would upstage disease and change treatment strategies.

### P19 Knowledge of and attitudes towards research activity within different staffing groups in a DGH Breast Unit

#### **Dr Simon Lowes**^**1,2**^, Lucy Blackwell^1^

##### ^1^Gateshead Health NHS Foundation Trust, ^2^Newcastle University

*Breast Cancer Research* 25(S1): P19

Clinical research is essential for improving the quality of healthcare in an evidence-based manner. Those most heavily involved with conducting research tend to be those delivering clinical care. Given its importance in making advances across the whole diagnostics and treatment pathway, there is scope to widen participation in research at different staff levels.

Within our breast unit, based in a District General Hospital and providing screening and symptomatic services, a 14-question survey was given to different staff groups investigating their awareness of research activities within the unit, attitudes towards research, and their level of involvement.

There were 59 responses from 7 consultant radiologists, 6 consultant surgeons, 10 breast care nurses (BCNs), 3 advanced practitioner radiographers, 10 radiographers, 8 assistant practitioners, 7 radiology healthcare assistants, and 8 admin staff.

Most staff were aware that research was carried out in the unit, and all radiologists, surgeons, and BCNs could name or describe at least one active study. Those who felt they were actively involved in research comprised mainly radiologists and surgeons, but overall most of the staff would like to be made more aware of research in the unit, and if given the opportunity would like to become involved with research. Almost all staff felt that research-active departments overall have a better reputation than non-research-active departments.

The findings overall demonstrate an appetite within all staffing groups to become more aware of and involved with research; encouraging participation at all levels may help to generate more engagement when conducting studies within breast units.

### P20 Patient preferences of contrast-enhanced spectral mammography (CESM) versus breast MRI

#### Magdalena Berezka-Zelek^1^, Lisa Snowdon^1^, **Dr Simon Lowes**^**1,2**^

##### ^1^Gateshead Health NHS Foundation Trust, ^2^Newcastle University

*Breast Cancer Research* 25(S1): P20

Contrast-enhanced spectral mammography (CESM) continues to gain momentum in the UK as an effective supplementary modality to investigate breast cancer. Its potential applications overlap those with breast MRI and for some indications may serve as an equivalent alternative. The aim of this study was to evaluate patient preference between the two modalities.

Patients undergoing both modalities for the same indication completed a questionnaire about their experiences of each procedure including the comfort of breast compression, the sensation of intravenous contrast, their level of anxiety, and overall their preference. Participants were also given the opportunity to explain their preference by free text. The first 70 consecutive patients returning their questionnaires were included.

Patients overall preferred CESM to MRI (63% versus 11%), with 26% having no preference. Although patients tended to find breast compression less comfortable during CESM, patients were overall more relaxed during CESM compared to MRI. No differences between modalities were found regarding patient comfort during intravenous contrast injection. Free text comments indicated that less noise, reduced anxiety and a shorter procedure time, all contribute toward a greater patient experience with CESM compared to MRI. Claustrophobia was frequently cited as a problem during MRI.

Should further studies evaluating the diagnostic capabilities of the two modalities support CESM as a replacement MRI for certain indications, this dataset confirms that CESM is well-tolerated and is overall preferred by patients compared to MRI. CESM may also be appropriate for patients where MRI is contraindicated or when claustrophobia is a problem.

### P21 Clinical outcomes of patients presenting with breast pain to one stop clinics over a two-month period

#### Dr Louise Littlewood^1^, Dr Sarrah El Tahir^1^, Dr Robert Strong^1^, **Dr Simon Lowes**^**1,2**^

##### ^1^Gateshead Health NHS Foundation Trust, ^2^Newcastle University

*Breast Cancer Research* 25(S1): P21

One-stop breast services are under relentless pressure caused by increasing referrals and insufficient staffing numbers. Although referrals to these clinics are made under the cancer two-week-wait pathway, the vast majority of patients have benign or normal findings. Breast pain is a very common reason for referral and is rarely associated with malignancy.

This study aimed to assess the outcomes of women presenting with breast pain over a 2-month period (March–April 2021). Proformas were completed by radiologists in clinic documenting the patient’s presenting symptoms, GP findings, surgical team findings, imaging requested, and outcomes.

Of 791 proformas completed, 184 women (23%) presented with breast pain as their only symptom. Mean age was 52 (median 52, range 17–93). Seventy-nine percentage (145/184) were aged 40+; of these, 133 had mammography on the day, with the remaining 12 having had previous mammograms < 12 months ago. Sixty-eight of 133 patients had mammography as their only investigation, and 77 had US in addition to mammography (including the 12 with mammography < 12 months earlier). All patients aged < 40 had US only (39/184).

In all 184 patients, only one cancer was found (0.54%), which was an incidental finding on mammography in the contralateral breast to the pain (age 79, G2 8 mm IDC, node negative).

This supports recent findings that breast pain alone is rarely associated with breast cancer, and imaging all women, particularly with US as an adjunct, appears not to be an efficient use of resources. Further stratification based on risk may help to refine the investigation pathway.

### P22 Perfusion estimates from DCE-MRI allow differentiation of pathological response categories in breast cancer patients undergoing neoadjuvant chemotherapy

#### **Dr Roido Manavaki**^**1**^, Dr Julia C Carmona-Bozo^1^, Dr Fatemeh Darvizeh^1^, Dr Muzna Nanaa^1^, Dr Gabrielle C Baxter^1^, Dr Ramona Woitek^1^, Dr Otso Arponen^1^, Dr Reem Bedair^1^, Dr Elena Provenzano^1^, Professor Martin J Graves^1^, Professor Fiona J Gilbert^1^

##### ^1^University of Cambridge, Cambridge, UK

*Breast Cancer Research* 25(S1): P22

Introduction: Dynamic contrast-enhanced (DCE)-MRI can monitor treatment response in breast cancer patients undergoing neoadjuvant chemotherapy (NACT). This work investigates whether changes in the DCE-MRI perfusion parameter Ktrans can differentiate pathological response categories in breast cancer patients undergoing NACT.

Methods: Women > 18 years receiving NACT before surgery for breast cancer underwent DCE-MRI examinations at 3 T at baseline, post-cycle 1, mid-treatment, and end-of-treatment. DCE-MRI series were analysed using the extended Tofts’ model to derive mean tumour Ktrans. Pathological response was classified using the Pinder response criteria: 1i = pathological complete response (pCR) without ductal carcinoma in situ (DCIS); 1ii = pCR including DCIS; 2i =  < 10% residual tumour; 2ii =  < 10–50% residual tumour; 2iii =  > 50% residual tumour; 3 = no response. Mixed effect models with random intercepts and slopes for subjects were used to study changes in Ktrans among pathological response groups during treatment.

Results: Data from 156 patients (53 ± 11 years) with 163 lesions were analysed. The majority were ductal carcinomas (136/163; 83%), hormone receptor-positive (96/163; 59%), with 66% (108/163) HER2-negative. Tumours were grade 2 or 3. Out of 163, 58 (37%) lesions showed pCR. There was a significant decrease in Ktrans per NACT cycle (− 15.4 ± 2.2%, p < 0.001), which was independent of tumour grade (p = 0.29) and molecular subtype (*p* = 0.49). Cancers displaying pCR (1i-1ii) showed a larger reduction in Ktrans than those with partial (2i–2iii) or no response (3); complete vs partial vs no response: − 13.2 ± 5.7% versus − 5.8 ± 3.5% versus − 0.3 ± 6.0% change/cycle; *p* = 0.03.

Conclusion: DCE-MRI perfusion estimates can differentiate pathological response categories in breast cancer.

### P23 Tips and Tricks for Assessment and Second Look Ultrasound in Detecting lesions seen on High Risk Screening MRI

#### **Dr Sarah Mcwilliams**^**1**^, Dr Sharon Koo^1^

##### ^1^South West London Breast Screening, St George’s Rose Centre, St George’s NHS Foundation Trust, Tooting, UK

*Breast Cancer Research* 25(S1): P23

Very-high-risk breast screening with MRI has created a complex group of women who can be recalled to the assessment clinic. The assessment may be performed by clinicians inexperienced with reporting breast MRI and the complexities of second-look ultrasound and MRI correlation. Assessment is challenging in this subgroup where in women under 40 there is no mammography for correlation and ultrasound is more challenging breasts due to the greater prevalence of cysts and fibroadenomas.

We present useful assessment guidelines and tips and tricks that can be used by all clinicians for assessing women recalled from Very High Risk Screening. The indications for mammography in under 40 and the use of additional mammographic imaging with Tomosynthesis will be discussed. Tips to aid MRI correlation with conventional imaging will be presented. For second-look ultrasound tips of use of 18 MHz and 14 MHz probes, Doppler, harmonics, shear wave, microvascular flow and focal point will be presented. Shear wave as an addition in second-look ultrasound aids detection of lesions seen on MRI. Ultrasound is a subjective technique, and it can be difficult to determine whether a lesion is real or a normal anatomical structure. We present examples of cases where the use of harmonics, shear wave, microvascular flow, the changing of the probe settings and probe frequency aid lesion detection.

We present a simple algorithm for Assessment of Very High Risk Women.

### P24 Tips and Tricks of Lateral Arm Stereotactic Biopsy in failed upright stereotactic biopsy or mammographically difficult cases

#### **Dr Sarah Mcwilliams**^**1**^, Dr Sharon Koo^1^

##### ^1^South West Thames Screening Programme, St George’s NHS Foundation Trust, Tooting, United Kingdom

*Breast Cancer Research* 25(S1): P24

The increasing resolution of digital mammography and introduction of tomosynthesis and tomosynthesis biopsy have improved lesion detection creating a need for sampling smaller and more challenging lesions. The shift to performing vacuum-assisted excision biopsy following B3 histology has further contributed to the demand for biopsying and excising more difficult lesions. The need for adapting biopsy technique and having flexibility in approach with increasing use of stereotactic vacuum excision will be presented.

The advantages of lateral arm stereotactic biopsy vs upright stereotactic biopsy and lateral arm’s advantage in mammographically difficult cases will be presented. At our institution, both upright and lateral arm biopsy can be performed.

Lateral arm biopsy is useful in performing stereotactic biopsy in the presence of breast implants using the Eklund technique, in sampling medial, lateral, inferior and posterior lesions or in very thin breasts when there is risk of trauma to the skin. The advantage of lateral arm biopsy visualising calcification in the biopsy chamber using the 3 o’clock and 9 o’clock positions will be discussed and how this can limit the number of cores taken decreasing the risk of haematoma. Examples of failed upright stereotactic biopsy and the benefit of Lateral arm and case selection aids will be presented. Tips and tricks and a flow chart aiding selection of Lateral arm cases will be discussed.

### P25 Automated breast density measurement comparison between reader Visual Analogue Scale (VAS) scores and two artificial intelligence algorithms

#### **Dr Nicholas Payne**^**1**^, Dr Sarah Hickman^1^, Richard Black^2^, Oliver Morrish^2^, Prof Fiona Gilbert^1,2^

##### ^1^University of Cambridge, ^2^Cambridge University Hospitals

*Breast Cancer Research* 25(S1): P25

Purpose: To compare two artificial intelligence (AI) algorithms in the assessment of mammographic breast density with Visual Analogue Scale (VAS) converted to Breast Imaging-Reporting and Data System (BIRADS) classification from two readers.

Methods: The TOMMY dataset includes raw and for presentation mammograms of women aged 40–73 years and breast density score on a VAS from one or two readers with at least two years of experience. The VAS score was converted to a BIRADS classification by taking thresholds; *a* = VAS < 25%, *b* = 25% ≤ VAS < 50%, *c* = 50% ≤ VAS < 75%, and *d* = VAS ≥ 75%.

Data from one of the six study sites were excluded as some of the cases had been used to train one of the AI algorithms. Cases contained one of each view (right/left cranial caudal/mediolateral oblique) in both raw and for presentation forms and were processed by two AI algorithms. Only cases with a BIRADS classifications from a reader, AI-1, and AI-2 were included in analysis (*N* = 2808). The algorithms AI-1 and AI-2 used raw and processed full-field digital mammography data, respectively.

Results: The linearly weighted Cohen’s kappa (κ) was calculated for each pairing. For readers and AI-1, κ = 0.35 showing fair agreement. For readers and AI-2, κ = 0.45 showing moderate agreement. For AI-1 versus AI-2, κ = 0.63 showing substantial agreement.

Conclusion: The AI-2 algorithm working on processed data is more likely to agree with a readers’ VAS score converted to the BIRADS classification. However, there is inter-reader variability in classification making it difficult to use reader scoring as the ground truth. The stronger correlation is seen between the two algorithms.

### P26 Impact of imaging on surgical outcomes in DCIS—a single centre experience

#### Dr Matthew Muller^1^, Dr Roisin Bradley^1^, Dr Isabelle Hanson^1^, Jenny Piper^1^, Jia Mang Lee^2^, **Dr Bhavani Rengabashyam**^**1**^

##### ^1^York and Scarborough Teaching Hospitals NHS Foundation Trust, York, United Kingdom, ^2^Hull York Medical School

*Breast Cancer Research* 25(S1): P26

Aim: To determine the correlation between radiological sizing and pathological sizing of ductal carcinoma in situ (DCIS) and its effect on breast conserving surgery (BCS).

Materials and Methods: Single-institution retrospective observational study. Three hundred and seventy-six DCIS cases identified from hospital electronic patient records between April 2015 and March 2021. One hundred and fifty-seven underwent BCS. Age, route of presentation, mammographic density, presence of calcification, imaging size, histological grade, preoperative localisation type, presence of specimen X-ray and cavity shaves noted. Concordance defined as preoperative imaging and surgical excision pathology size correlation within 5 mm, underestimation/overestimation as divergence by 5 mm or more. Statistical analysis performed using Student’s *t*-test and Chi-square.

Results: Median age 60 years (37–87 yrs). 139 (88.5%) screen-detected cases; 142 (90.4%) had mammographic calcification. 111 (70.7%) had high-grade DCIS, 89 (56.6%) had comedonecrosis.

Out of 157, 101 (64.3%) underwent single surgery, 56 (35.6%) had re-excision. Out of 56, 19 (33.9%) had mastectomy. Sixty-two (39.5%) concordant, 55 (35%) underestimated and 40 (25%) overestimated. Out of 55, 35 (63%) underestimated cases underwent a second surgery, 47/62 (75%) concordant cases had single surgery (*p* = 0.03). Out of 157, 116 (73.8%) cases measured < / = 30 mm on final histology. In DCIS < / = 30 mm, there was concordance between radiology and pathology (median 1 mm). In DCIS > 30 mm, imaging underestimated size (median 16 mm), (*p* < 0.05). Average final pathology size in single versus re-excision cases was 17.25 mm and 33.02 mm, respectively (*p* < 0.05). One hundred and fifty-two women (96.8%) had preoperative localisation.

Conclusion: This study suggests imaging underestimates DCIS > 3 cm resulting in positive margins. Prospective studies are recommended to confirm findings and to identify factors that could reduce the sizing mismatch.

### P27 Wireless Localisation: Audit of our Local Experience, Planned Future Developments and Projected Efficiencies.

#### **Dr Jennifer Royds**^**1**^, Dr Gauripriya Babu^1^, Gavin Loy^1^, Dr Alexandru Calciu^1^

##### ^1^NHS Lothian, Edinburgh, UK

*Breast Cancer Research* 25(S1): P27

Introduction: We present our audit of 238 wireless localisations, also patient feedback results. We outline our transformational plans for localisation at recall assessment and the potential resource efficiencies/patient benefits we have extrapolated from our data.

Results:

Localisation audit:

101 saviscout, 137 magseed excisions.

128 were screen detected.

Overall re-excision rate—16.8%, 14.8% for screening cases.

Patient experience was positive.

One hundred and two (43%) met proposed suitability criteria (unifocal, unequivocally malignant) for localisation at recall assessment.

Localisation of appropriate lesions at screening recall biopsy will commence in August 2022. We extrapolate the following:

323 localisations annually would be appropriate for localisation at recall biopsy.

Symptomatic US appointment savings (15 min per localisation): 81 h.

Vetting/reviewing of localisation requests (10 min per case): 54 h.

(Overall saving of 135 DCC hours of consultant time).

Radiographic post-insertion film acquisition time saving (15 min per case): 81 h.

A&C appointing/booking time (15 min per case) 81 h.

Cost-savings of markers (30.87 per marker): £9971.01.

Three hundred and twenty-three patients would be spared a second procedure, remote from their home address, with risk of confusion over localisation and operative date eliminated.

In September 2022, appointments previously allocated to localisations at our institution will be reallocated to new patient imaging generating 30 weekly slots (a full NPC) of extra imaging capacity with no additional resource.

Conclusion: Wireless localisation is an effective and acceptable localisation method. Localisation at recall biopsy presents an opportunity for significant efficiency gains, marker cost savings and patient benefits.

### P28 Evaluating the Evolution of Paediatric Breast Referrals in North East London

#### **Dr Sonali Shah**^**1**^, Dr Essam Lakha^1^, Dr Tamara Suaris^1^

##### ^1^Barts Health NHS Trust, London

*Breast Cancer Research* 25(S1): P28

The number of patients aged less than 18 years that were referred to the BartsHealth paediatric breast radiology service were reviewed over 7.5 years from 2015–2022. This was across 4 centres- Royal London Hospital (RLH), Whipps Cross Hospital, St Bartholomew Hospital and Newham General Hospital, providing coverage for most of North East London.

Between 2015 and 2022, a total of 423 patients were referred to this service. There has been a yearly incremental increase in the number of patients assessed with 31 patients referred in 2015 and 103 patients in 2022. Of the total number of patients, the most common age group was 14–18 years. Over the 7.5-year period, the growth in volume is largely driven by the 14–18 age group and the 8–14 age group. The youngest (< 8 years) group has not significantly increased over the past 7.5 years with an average of 8 patients/year.

The percentage of patients in each age bracket per year demonstrates that the oldest cohort (14–18) has increased as a proportion of the total from 50% in 2015–2018 to 60% + from 2019 onwards. The younger age groups display more volatile trends, however the small sample size limits our ability to draw reliable conclusions. However we do note that the < 8 years cohort has reduced as a percentage of total from an average of 23% in the 2015–2018 period to 12% in 2018–2022.

We will demonstrate how the dedicated paediatric breast clinic may have changed referral practice and also demonstrate paediatric breast surgical outcomes following referrals.

### P29 Little Person Breast Us Audit: Less Is More. A retrospective 5-year review of breast ultrasound in adolescence and young children in a single service

#### Dr Elisabetta Giannotti^1^, **Mrs Rachel Sun**^**1**^

##### ^1^Nottingham University Hospital NHS Trust, Nottingham, United Kingdom

*Breast Cancer Research* 25(S1): P29

Introduction: While breast cancer is rare in adolescents and young adults (AYAs), breast symptoms are common. The aim of this study was to review if breast ultrasound can be safely omitted in AYAs to increase efficiency by reducing radiology workload.

M&M: A retrospective review of breast ultrasound (US) of AYAs < 19 years (2016–2020) was conducted. Data regarding presentation, clinical opinion, ultrasound, biopsy and surgical outcomes were collected. At least 18-month follow-up was achieved for every patient.

Results: No 586 (562 females, 24 males) AYAs, with a median age of 16.5 years, who had a breast US were included.

There were two cases of phylloides tumour (1 benign and 1 border line) (incidence 0.3%).

In females, the presenting symptoms were lump (89.6%), infection (4%), pain (3.3%), lump and skin changes (1%), nipple discharge (0.9%), axillary lump (0.9%) and trauma (0.3%).

Clinically 53% (298) were P1 and 47% (264) were P2.

Ultrasound findings were normal (55.5%), demonstrated a well-defined mass (36.6%), cystic lesion (4.8%), fluid collection (1.6%) or skin lesion (1.5%).

Six patients had ultrasound-guided biopsy (6 fibroadenoma) and 27 had surgical excision fibroadenoma (22), hamartoma (2), phylloides (2) and angiomixoma (1). All phylloides tumour were clinically palpable P2.

Conclusion: Ultrasound in AYAs could safely be omitted if clinically P1. This approach would have avoided breast ultrasound in 298 (53%) patients. Even if clinically P2 the risk of upgrade is low, there is no need to biopsy prior to surgical excision and the lesions could be managed clinically.

### P30 Male breast disease—what the radiologist needs to know

#### Dr Daniella Kostic^1^, **Dr Matthew Wallis**^**1**^, Dr Nuala Healy^1^

##### ^1^Addenbrooke's Hospital, Cambridge University Hospitals NHS Trust, Cambridge, United Kingdom

*Breast Cancer Research* 25(S1): P30

Introduction: Male breast disease encompasses both benign and malignant conditions, with gynaecomastia the most commonly encountered disease entity. Male breast cancer is rare, accounting for 1% of all breast cancer cases in the UK. Recognition and differentiation of malignancy from benign disease, such as unilateral gynaecomastia, are essential for early detection and improved outcomes. Imaging guidelines for assessing symptoms in males are variable, thus resulting in an inconsistent imaging work-up.

The aim of this review is to illustrate the imaging features of male breast disease, with discussion of appropriate imaging of symptomatic males.

Method: A retrospective review was undertaken of male patients attending the Cambridge Breast Unit. Patient electronic records and imaging were reviewed for clinical history, imaging findings, biopsy results and patient outcomes.

Results: This educational review highlights guidelines on when to perform breast imaging in males attending symptomatic services, with particular reference to type of imaging. Illustrative cases of male breast disease are included to demonstrate classic mammographic and sonographic appearances of benign and malignant disease. Examples of malignancy, invasive carcinoma of no special type and papillary carcinoma will be included. In this review, there is specific emphasis on gynaecomastia, the most common benign condition, with imaging examples of the various stages. This educational review also contains representative examples of other benign male chest wall conditions such as hidradenoma, epidermal inclusion cysts, haemangioma and angiolipoma.

Conclusion: Familiarity with imaging features of male breast disease allows more accurate imaging interpretation, as well as avoidance of unnecessary procedures and treatment.

### P31 Survey of Irish radiology registrars: Despite lack of exposure, are trainees interested in Breast Radiology?

#### **Dr Ivan Welaratne**^**1**^, Dr Niamh O’Mahony^1^

##### ^1^Mater Misericordiae University Hospital, Dublin 7, Ireland

*Breast Cancer Research* 25(S1): P31

Purpose: With an ever-increasing workload, there is more demand for permanent radiology consultant posts, particularly in breast radiology. Each year, only a limited number of training posts for breast radiology are provided. A national survey of Irish trainees was created to analyse interest in breast radiology.

Materials and Methods: A questionnaire was devised for the trainees containing a total of 14 questions. It assessed demographics, followed by subspecialty interests and ending with direct questions relating to interest in breast radiology.

Results: Across nine training sites, 52 completed responses were returned from a total of 131 trainees (40%). Thirty-one trainees were in years 1–3 (60%). Seventy-nine percentage had yet to complete a breast rotation, with most centres not providing dedicated sessions. Sixty-five percentage had decided on subspecialty interests, with only 4 (10%) having breast as their first choice. Interventional and musculoskeletal were the most popular first choices. Fifty percentage would consider breast with the main reasons being mix of diagnostic/procedures, upcoming jobs and predictable workload. Most of the respondents not considering breast felt increased exposure during training probably would have made a difference.

Conclusion: There is a positive outlook towards breast radiology in Ireland. However, the main limiting factor for increasing interest is lack of dedicated sessions during general training. This should be mandated as part of training as it is an important part of radiology, especially with regard to screening.

### P32 Can we avoid biopsy in sonographically benign solid breast masses with a soft shear-wave elastography value?

#### **Dr Lara Maria Zammit**^**1**^, Dr Reena Aggarwal^1^, Julie Smith^1^, Dr Miaad Al-Attar^1^

##### ^1^University Hospitals of Leicester

*Breast Cancer Research* 25(S1): P32

Introduction: Solid breast masses in patients < 25 years with benign grey-scale ultrasound imaging (U2) do not need biopsy. Patients over this age, irrespective of sonographic morphology, will have biopsy in our unit. Shear-wave elastography (SWE) has proven to be accurate in differentiating benign and malignant masses, with a cut-off of 50 kPa suggested in the literature.

Aim: We investigate whether it is safe to avoid biopsy in patients with a benign breast mass and soft SWE (< 50 kPa).

Method: Retrospective search of patients who had SWE between 2021 and 2022. The ultrasound grading, SWE value and histology were recorded.

Results: Our study included 190 patients aged 17–89 years (mean age 47). One hundred and ninety-three lesions were analysed; 106 (55%) benign and 87 (45%) malignant on histology. One hundred and thirty-two lesions had a hard SWE, of these 80 (60.6%) were malignant. Sixty-one lesions had a soft SWE, of these 7 (11.5%) were malignant (false negatives)—all of were graded U3 and above. Fifty-four lesions (28%) were graded U2. Of these, 30/54 (56%) had a soft SWE and all of them were benign on histology. The remaining 24/54 had a hard SWE with one of them being malignant. Overall, the sensitivity of SWE is 92% with a specificity of 50.9%.

Conclusion: We can avoid biopsy in benign breast masses with a soft SWE. We should sample lesions with a hard SWE even if sonographically benign. Lesions with a higher ultrasound grading (U3 and above) need sampling irrespective of the SWE value.

## Footnotes

1 10.1007/978-3-031-12053-4_3.

2 10.1007/978-3-031-12053-4_3.

3 10.1002/mrm.29076.

4 10.1007/978-3-031-12053-4_3.

